# Post‐association barrier to host switching maintained despite strong selection in a novel mutualism

**DOI:** 10.1002/ece3.9011

**Published:** 2022-06-17

**Authors:** Zoe M. Dinges, Raelyn K. Phillips, Curtis M. Lively, Farrah Bashey

**Affiliations:** ^1^ Department of Biology Indiana University Bloomington Indiana USA

**Keywords:** evolutionary rescue, host switching, mutualism, post‐association barrier, *Steinernema*, *Xenorhabdus*

## Abstract

Following a host shift, repeated co‐passaging of a mutualistic pair is expected to increase fitness over time in one or both species. Without adaptation, a novel association may be evolutionarily short‐lived as it is likely to be outcompeted by native pairings. Here, we test whether experimental evolution can rescue a low‐fitness novel pairing between two sympatric species of *Steinernema* nematodes and their symbiotic *Xenorhabdus* bacteria. Despite low mean fitness in the novel association, considerable variation in nematode reproduction was observed across replicate populations. We selected the most productive infections, co‐passaging this novel mutualism nine times to determine whether selection could improve the fitness of either or both partners. We found that neither partner showed increased fitness over time. Our results suggest that the variation in association success was not heritable and that mutational input was insufficient to allow evolution to facilitate this host shift. Thus, post‐association costs of host switching may represent a formidable barrier to novel partnerships among sympatric mutualists.

## Graphical abstract

A novel pairing of Steinernema nematodes and Xenorhabdus bacteria was copassaged for 9 generations. Despite strong selection, the pairing did not demonstrate fitness improvement relative to highly fit native pairs. These results suggest that barriers preventing host switching in this system may be difficult to overcome.

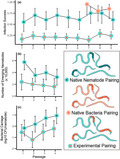

## 1 INTRODUCTION

Specialization, which is frequently observed in mutualistic interactions (Chomicki et al., 2020), can make host switching costly. These costs can manifest as lower growth, fecundity, or survival for hosts partnered with non‐native symbionts (Chapuis et al., 2009; Ehinger et al., 2014; Parker, 1995; Sicard et al., 2005). Analogously, symbionts may be less competent in colonizing a novel host, and thus, less likely to be transmitted across hosts (Kwong et al., 2014; Sicard et al., 2005). When costs are severe, the partners must rapidly adapt in order for a new mutualism to be successful. Serial co‐passaging studies have shown that adaptation can occur very rapidly (Batstone et al., 2020; Robinson et al., 2018; Shapiro & Turner, 2018; Soto et al., 2012). However, even with strong selection, evolution could be constrained by low standing genetic variation or the relative strength of genetic drift in small populations (e.g., Castillo & Delph, 2016; Hoang et al., 2016; White et al., 2021). Additionally, if changes across multiple loci are required to adapt to a new host, increases in fitness may take longer to arise (Streicker et al., 2012), and may limit the evolutionary success of a new partnership in nature. Here, we examine the response to strong selection following an experimental host shift between sympatric isolates of the *Steinernema* nematode–*Xenorhabdus* bacteria mutualism.

In the entomopathogenic mutualism between *Steinernema* nematodes and *Xenorhabdus* bacteria, nematodes transmit bacteria between insects, and bacteria help kill and digest the insect. The bacteria also help to reduce competition by producing toxins against non‐native nematodes and other co‐infecting microbes (Bashey et al., 2013; Murfin et al., 2018). In a previous study, we paired nematodes with symbiotic bacteria isolated from other sympatric nematode species (Dinges et al., 2022). This experiment focused on a single bacteria species, *X. bovienii*, isolated from each of three *Steinernema* nematode species. Symbionts faced no barriers to host switching within nematode species or between closely related nematode species. However, we observed strong barriers to host switching when nematode species were more distantly related. Specifically, *S. kraussei* paired with any strain of *X. bovienii* isolated from clade III nematodes (*S. kraussei* or *S. texanum*) showed no significant differences in fitness relative to the native pairing, while pairings across nematode clades were reciprocally unsuccessful. In fact, only the pairing used in this study (out of 10 attempted) had any infection success once associated (Dinges et al., 2022). The newly associated partners were able to successfully infect insects and reproduce, albeit at a reduced probability and lower fecundity. This post‐association barrier to host switching is predicted to limit the spread of this novel combination in nature. Thus, although this host switch is possible, the post‐association barriers result in partner fidelity feedback favoring the native pairing (Murfin et al., 2015; Sachs et al., 2004). Nevertheless, rapid evolution could rescue this otherwise unhopeful pairing allowing for a host shift.

In this study, we tested whether evolution could facilitate a host shift by experimentally passaging the novel pairing to see if it could respond to strong selection. In each of the nine passages through insects, we selected the most fecund of the novel pairings to propagate the mutualism. We assessed the fitness of the mutualism with three metrics: the proportion of successful infections, the mean number of nematodes emerging from successful infections, and the mean number of bacterial cells carried per nematode. We predicted that if the observed variation in fitness was heritable, the novel combination should evolve, exhibiting increased fitness across subsequent passages as the partners adapt to each other. Instead, we found that none of our metrics of fitness improved over time. We suggest that low genetic variation, and repeated bottlenecks in the mutualism, may constrain the short‐term response to selection.

## 2 METHODS

### 2.1 Pairings

We experimentally paired aposymbiotic (lacking symbionts) *Steinernema kraussei* nematodes with *Xenorhabdus bovienii* bacteria cultured from *S. affine* nematodes (Table 1) as described in Dinges et al. (2022). These nematode stocks originated from soil samples collected from the same hillside at the same time, approximately 60 m apart from each other (see Bertoloni Meli & Bashey, 2018). The bacteria strain was isolated from a single colony obtained by crushing the first lab‐reared batch of juvenile nematodes after field collection (Bertoloni Meli & Bashey, 2018). The experimental pairing exhibited reduced infection success and reduced nematode emergence compared to the native pairing. Despite these costs, the experimental pairing exhibited bacterial carriage similar to the native pairing (Dinges et al., 2022). Here, we examine whether infection success and nematode emergence would respond to strong selection from repeated co‐passaging. The experimental pairing was compared to the native nematode control group, *S. kraussei*, and the native bacteria control group, *S. affine* (Table 1). Both control groups carried their native bacteria, having never been cultured separately.

**TABLE 1:** Nematode and bacteria for each pairing

Pairing	Nematode species (Stock)	Bacteria strain	Nematode GenBank accession #s	Bacteria genome Accession #s
Native nematode control	*S. kraussei* (MC 239)	*X. bovienii* from MC 239	OK319049 OK305943	JAILSW000000000
Native bacteria control	*S. affine* (MC 226)	*X. bovienii* from MC 226	OK319044 OK305939	JAILSS000000000
Experimental pairing	*S. kraussei* (MC 239)	*X. bovienii* from MC 226		

### 2.2 Passaging

**FIGURE 1:**
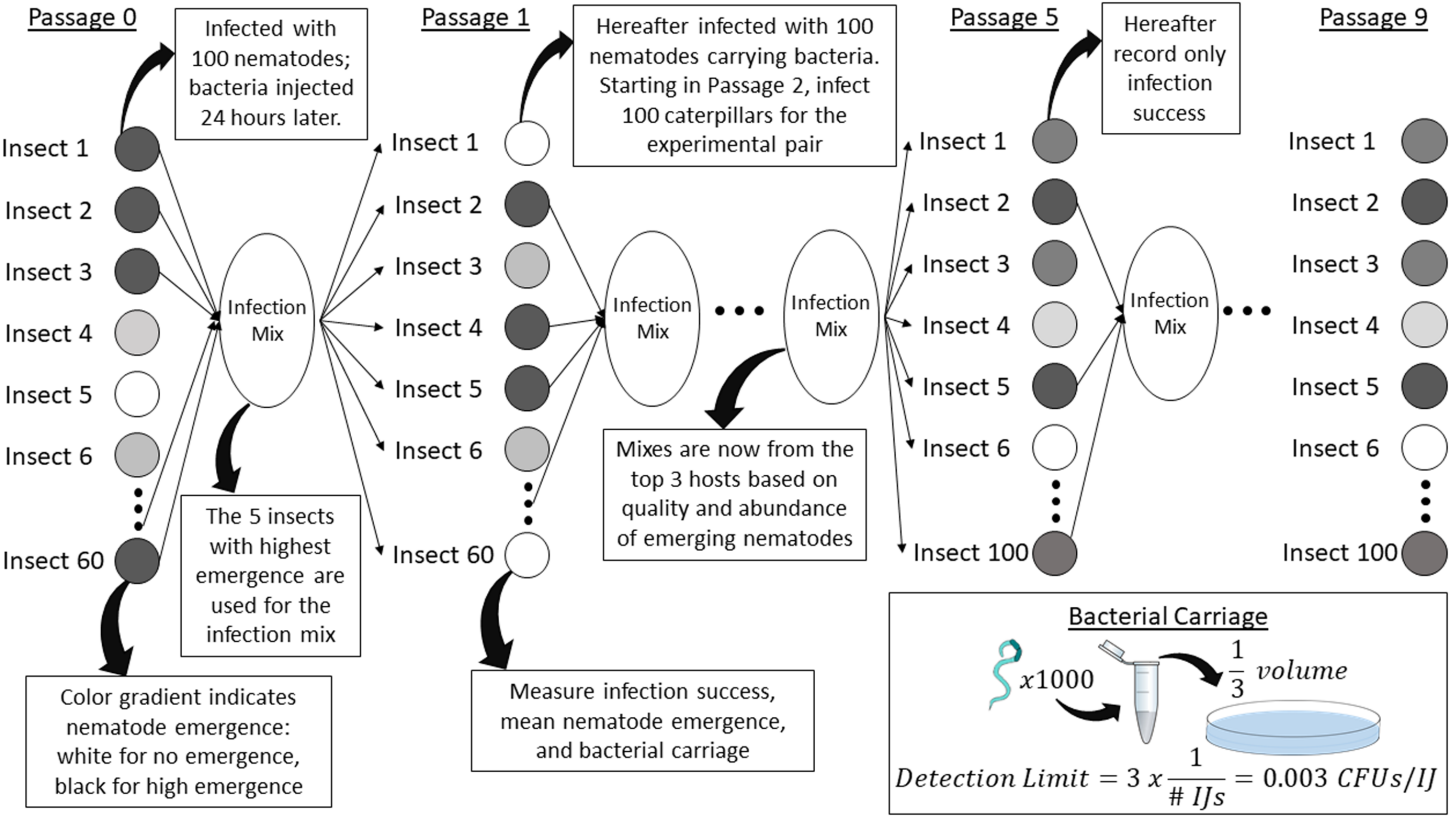
Schematic of the passaging protocol and fitness components measured for each nematode–bacteria pairing. Starting in passage 5 to lessen the effort needed to maintain the experimental lines, and to increase the selection on successful pairings, infection mixes were created by combining nematodes from the three best infections based on a visual inspection of the quality and abundance of emerging nematodes. In addition, only infection success was measured

All nematode–bacteria pairings were passaged (Figure 1) through *Galleria mellonella* caterpillars by pipetting 100 nematodes (carrying bacteria) in 500 μl of ddH_2_O onto the dorsum of the caterpillar as described in Bashey and Lively (2009). These caterpillars were kept in individual infection arenas consisting of a 60 × 20 mm petri dish with a 55 mm filter paper. In each passage of the two native controls, we infected 20 caterpillars. For the experimental pairing, we infected 60 caterpillars in passage 1, and increased this to 100 caterpillars for the remaining passages due to low infection success (proportion of caterpillars with nematode emergence).

Caterpillar mortality was assayed within 7 days post‐infection; dead caterpillars were moved to modified White traps to allow for the emergence and collection of juvenile nematodes (Bashey & Lively, 2009). Emergence was assayed for a further 3 weeks, after which infective juvenile nematodes (IJs) were collected within 6 weeks post‐infection and kept in culture flasks at 4°C. Infection success was measured as the proportion of infected caterpillars with any emerging nematode progeny. The number of emerging nematodes was estimated by volumetric subsampling. We estimated bacterial carriage by crushing a sample of 1000 IJs for five collections per pairing per passage. These nematodes were surface sterilized, crushed in PBS, and one‐third of the volume was plated on NBTA media (Nutrient Agar + 0.0025% bromothymol blue + 0.004% triphenyltetrazolium chloride; Akhurst & Boemare, 1988) and grown at 28°C for 48 h. Focal *X. bovienii* colonies exhibit a readily identifiable colony morphology on this media (Bashey et al., 2012). We used the colony counts (CFUs) from the plates to estimate the carriage per nematode. If no colonies grew from the crushing, we used the detection limit (equation in Figure 1 inset). Of 40 total samples, two had zero CFUs (Experimental Pairing Passage 2 and Experimental Pairing Passage 3). These zeros were replaced with the detection limit, 0.003 CFUs/IJ.

For passages 1–4, infection mixes were created by combining IJs from the five best infections of the previous passage based on the number of IJ emerging with respect to caterpillar mass. Starting in passage 5, to lessen the effort needed to maintain the experimental lines, and to increase the selection on successful pairings, infection mixes were created by combining nematodes from the three best infections based on a visual inspection of the quality and abundance of emerging nematodes. The bacteria control was introduced in passage 7 from a laboratory stock maintained for six prior passages in the lab. In addition, for passages 5–9, only infection success was measured.

### 2.3 Statistical analyses

All statistical analyses were performed using generalized linear models in R version 3.6.3 (R Core Team, 2020). We used the dplyr package to organize the data, and the ggplot2 package for all graphs (Wickham, 2016; Wickham et al., 2020). Differences in infection success (i.e., the proportion of caterpillars with nematode emergence) between the experimental and native control pairings were analyzed with logistic regression by specifying a binomial response variable for each caterpillar and testing the effect of pairing, passage, and their interaction. The number of emerging nematodes from each successful caterpillar and their mean bacterial carriage (log‐transformed) were each separately analyzed via ANOVA assuming a normal distribution with pairing, passage, and their interaction as main effects. For each model, we computed the estimated marginal means and 95% confidence intervals using the emmeans package (Lenth, 2020) and the *F* statistics for each independent variable using the rstatix package (Kassambara, 2020). Note that each pairing was represented by one evolutionary lineage, so confidence intervals reflect variation across insect hosts. Mean trait values for each pairing encompass both genetic and environmental changes with time. We focused on the interaction between pairing and passage in order to test whether the experimental pairing shows any evidence for adaptation in response to co‐passaging. This approach uses the nematode control pairing as a benchmark for a well‐adapted mutualism.

## 3 RESULTS

### 3.1 Infection success

**FIGURE 2:**
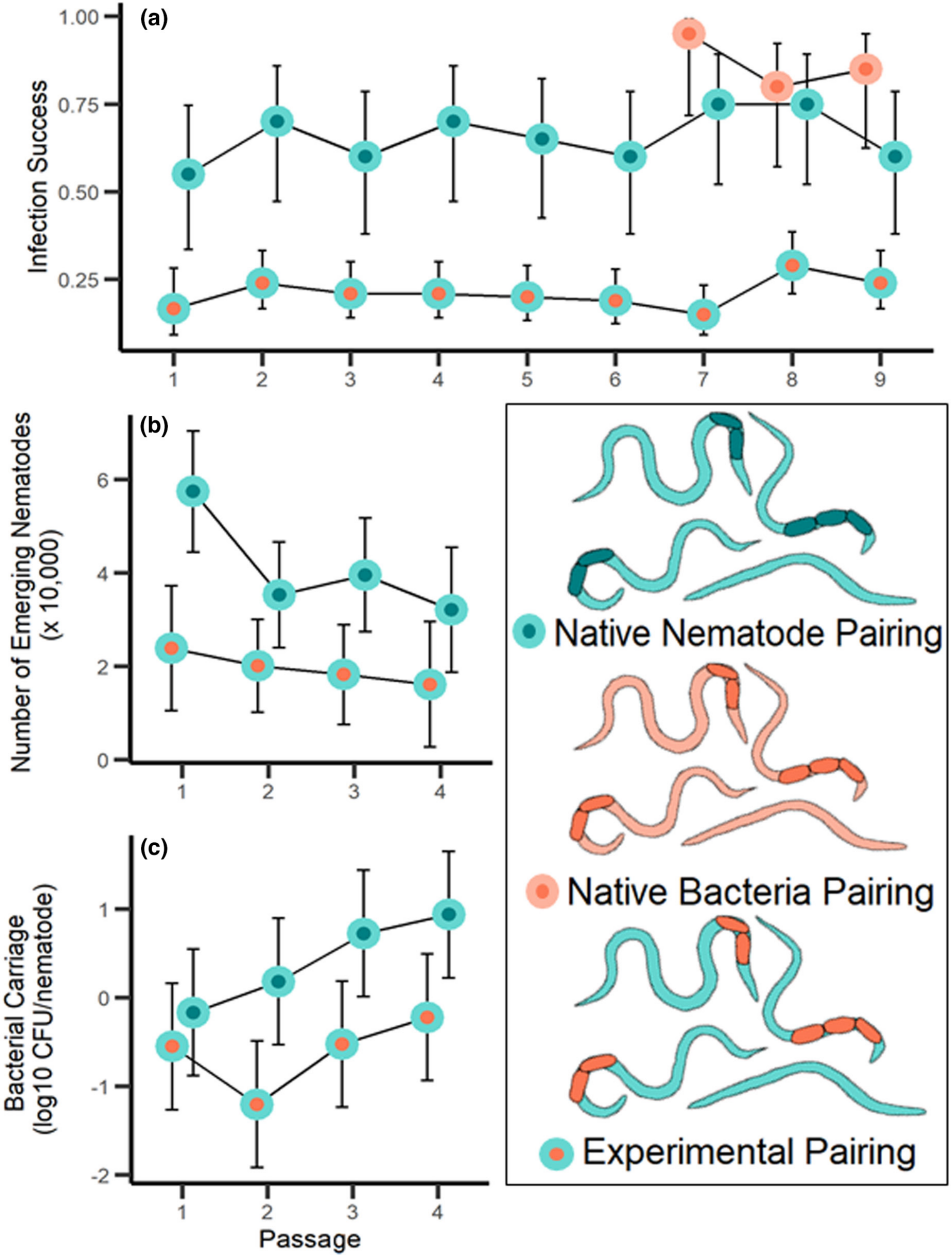
(a) Mean infection success (proportion of caterpillars with any nematode emergence) of each nematode pairing across nine passages. (b) Mean number of juvenile nematodes that emerged from successful infections and (c) the mean number of bacteria cells carried per nematode (log_10_ transformed). The log_10_ axis ranges from 1 (10 bacteria cells per nematode) to −2 (1 bacteria cell per 100 nematodes). All error bars are 95% confidence intervals around the mean. Blue circles with orange centers are the experimental pairing of *S. kraussei* nematodes carrying bacteria from *S. affine*, blue circles are *S. kraussei* nematodes carrying their native bacteria, and orange circles are *S. affine* nematodes carrying their native bacteria. Note the native bacteria pairing was maintained separately in the lab, and was only added to this experiment in passage 7 to test whether *S. affine* bacteria could account for the low infection success seen in the experimental pairing. The bacteria in the inset diagram are not to scale

The experimentally associated pairing had significantly lower infection success than the native nematode control group (Figure 2a; *F*_1, 1022_ = 128.751, *p* < .001). While only 21% of the caterpillars infected with the experimental pairing produced progeny nematodes, an average of 66% of those infected with the native nematode control group did (Figure 2a). Surprisingly, infection success did not increase in the experimental pairing relative to the nematode control over time (passage*pairing interaction *F*_8, 1022_ = 0.425, *p* = .907, Figure 2a). This failure to improve in the probability of producing any progeny from an infected host indicates a lack of adaptation in a key component of fitness for the novel mutualism. Moreover, the addition of the native bacteria pairing in passages 7–9 shows that both controls had higher infection success than experimental pairing (Figure 2a; *F*_2, 411_ = 59.484, *p* < .001), indicating that neither partner individually is responsible for the lowered infection success; it is the pairing of both partners together which leads to poor infection success.

### 3.2 Number of emerging nematodes

Fewer nematodes emerged from successful infections in the experimental pairing than in the native nematode control (Figure 2b; *F*_1, 94_ = 24.081, *p* < .001). In fact, on average, the experimental group had approximately 50% fewer nematodes emerge from successful infections than the native nematode control. There was a significant effect of passage (Figure 2b; *F*_3, 94_ = 2.865, *p* = .041), as nematode emergence was higher in the first passage than in the subsequent ones. Such temporal variation is common as insect hosts are sourced commercially and shipped to the lab. Critically though, the experimental pairing did not improve with time relative to the control (pairing‐by‐passage interaction *F*_3, 94_ = 1.173, *p* = .324, Figure 2b). Thus, the novel partners in the experimental pairing showed no evidence for increasing in their reproductive fitness despite strong selection.

### 3.3 Bacterial carriage

Nematodes in the experimental pairing carried fewer bacterial cells than nematodes in the nematode–native control pairing (Figure 2c; *F*_1, 32_ = 17.791, *p* < .001). In the native pairing, each nematode carried approximately a single cell of bacteria. In contrast, there was approximately one cell per every 10 nematodes in the experimental pair. There was a marginally significant effect of passage number (*F*_3, 32_ = 2.635, *p* = .067), as overall bacterial carriage increased over time. However, there was not a significant interaction between pairing and passage number (Figure 2c; *F*_3, 32_ = 0.824, *p* = .491), indicating that bacterial carriage did not change in the experimental pair relative to the native nematode pair over successive passages. Thus, the partners in the novel mutualism were not more likely to associate after repeated co‐passaging.

## 4 DISCUSSION

Post‐association barriers to host switching can limit the reproductive success of novel pairings, especially when they face competition with native pairs. If partner fidelity affords significant fitness advantages, then novel pairings may be possible, but of little consequence (Murfin et al., 2015; Sachs et al., 2004). Evolution, however, has the potential to rescue (Bell, 2017) low fitness pairings. Here, we tested whether a post‐association barrier to host switching could be overcome by strong selection. We expected that one or both partners would adapt, leading to increasing fitness over successive passages. We found that none of the fitness measures exhibited a significant interaction between the mutualism pairing and passage number, indicating that the experimental pair did not change relative to the native pair(s) across the successive passages. Thus, post‐association barriers were maintained despite repeated co‐passaging. The lack of response to selection in our experiment suggests that host shifts in this system may be limited by a lack of additive genetic variation.

Because we passaged only the most fecund infections, and because unsuccessful infections have a fitness of zero for both partners, this experiment imposed very strong selection on both the nematode and on the bacteria. Despite the strength of selection, the experimental pair did not improve over the passages, suggesting low genetic variation in the nematode and bacteria. Large population sizes within the insect (nematodes >10^4^, bacteria >10^6^) allow for mutational input to occur in both species. Indeed, previous studies have shown that some *Xenorhabdus* traits (e.g., growth rate and bacteriocin production) can evolve when passaged with nematodes in the lab (Bhattacharya et al., 2019; Morran et al., 2016). However, it is possible that adaptation to new partners requires multiple mutations, which would constrain the speed of evolutionary rescue.

Several experiments indicate that expanding host ranges relies on the accumulation of multiple mutations, which is less likely, and requires more time, than a single mutation (Hall et al., 2011; Longdon et al., 2014; Meyer et al., 2012; Quides et al., 2021; Soto et al., 2019; Streicker et al., 2012; Woolhouse et al., 2005). Because we selected on nematode emergence, mutations that increase nematode survival or reproduction should be favored. However, changes in bacterial transmission would likely require different mutations. The presence of one type of mutation without the other could indicate a tradeoff which could undermine the mutualism. Tradeoffs between mutualism fitness metrics, such as bacteria growth within the insect and transmission, have already been reported in the *Steinernema–Xenorhabdus* system (Cambon et al., 2019).

Despite large population sizes within the insect, both the nematode and bacterial populations go through a dramatic transmission bottleneck. Not all of the nematodes used in an infective dose are expected to survive and reproduce (Selvan et al., 1993). Using 100 IJs as an infective dose, between 20 and 50 nematodes would make up the founding population for each infection, and using higher doses results in higher within‐host mortality (A. Ramesh, unpublished data). Other selection experiments using *Steinernema* nematodes have demonstrated that responses to selection can be dampened by low founding population sizes (Bashey & Lively, 2009; Stuart & Gaugler, 1996). In terms of the bacterial populations, we found that bacterial carriage in the experimental pairing was one‐tenth that of the native pairs (Figure 2c), which would further limit the evolutionary potential of the novel mutualism. Thus, repeated, severe bottlenecking likely lowered the chance that a beneficial mutant was transferred to the next infection. Without new genetic input, variation in infection outcome would depend mostly on environmental variation across caterpillars. This could be due to demographic stochasticity affecting bacterial carriage and nematode survival within each insect host, as well as differences in the insect's nutrient composition, microbiome, and immune response.

So, does the maintenance of the post‐association barriers we observed indicate that host switching is unlikely to be facilitated by evolutionary rescue? Not necessarily. Here, we attempted to evolve one lineage, but perhaps if we had passaged multiple lineages, one of the incipient pairings might have had the right mutation. Alternatively, a different source stock might have been more successful. However, the *X. bovienii* stocks isolated from *S. affine* are highly genetically similar and equally distant from the *X. bovienii* isolated from *S. kraussei*, so it is not obvious that another *S. affine* associate strain would be more likely to successfully shift hosts.

Alternatively, traits involved in host switching may be acquired by horizontal gene transfer rather than by mutational input. In our experiment, gene flow among *X. bovienii* strains was restricted; however, in nature, this possible source of genetic variation may facilitate the adaptation of a novel mutualism. In fact, genomic analyses indicate gene flow has occurred among *X. bovienii* bacteria associated with different nematode species in our source population (Papudeshi et al., 2022). However, the likelihood that the genes transferred facilitate host switching and whether the frequency of these transfers is high enough to allow a novel combination to adapt is currently unknown. Nevertheless, our data suggest that standing genetic variation and mutational input alone is unlikely to allow evolution to rapidly mitigate the costs of host switching in genetically distant symbiont pairs.

All together, we conclude that highly specialized, mutualistically dependent partners can form barriers to host switching. These barriers may be difficult to overcome, preventing novel mutualisms from becoming established in nature. While this was tested here with bacterial symbionts, these patterns could also hold true in other types of mutualisms, given at least one partner is an obligate specialist. Mutualistic dependence, in which there is a cost to losing a mutualism partner, paired with specialization, in which limited partner genotypes are acceptable, would strengthen the barriers to host switching (Chomicki et al., 2020). In addition, limited genetic variation would slow the adaptation to a novel mutualism, as seen in our system.

## AUTHOR CONTRIBUTIONS

**Zoe M. Dinges:** Conceptualization (equal); formal analysis (equal); investigation (lead); methodology (supporting); visualization (lead); writing – original draft (equal); writing – review and editing (equal). **Curtis M. Lively:** Conceptualization (equal); funding acquisition (equal); writing – original draft (equal); writing – review and editing (equal). **Farrah Bashey‐Visser:** Conceptualization (equal); formal analysis (equal); funding acquisition (equal); methodology (lead); writing – original draft (equal); writing – review and editing (equal).

## CONFLICT OF INTEREST

We have no conflict of interest to declare.

## Data Availability

The data (csv), code (Rmd), output (html), and a dataset description (README.txt) have been uploaded to Dryad: doi:10.5061/dryad.xd2547dj3
